# Awareness, Knowledge, Attitudes, and Practices Regarding Diabetic Retinopathy Among Residents of Jazan City, Saudi Arabia

**DOI:** 10.7759/cureus.71219

**Published:** 2024-10-10

**Authors:** Abdulaziz A Alagsam, Essam A Alhazmi, Osama A Mobarki, Mohammed E Mojiri, Ahmed Y Najmi, Elaf J Zurayyir, Fatimah M Akkam, Reham M Aljudayba, Razan M Jurebi, Remas F Koko, Waad K Najmi, Hani A Al-Ghamdi, Sawsan J Kreet, Renad A Madkhali, Hanen I Alsuri

**Affiliations:** 1 Ophthalmology, Prince Muhammed bin Nasser Hospital, Jazan, SAU; 2 College of Medicine, Jazan University, Jazan, SAU; 3 College of Medicine, Al-Baha University, Al-Baha, SAU

**Keywords:** diabetic retinopathy, eye health, health education, jazan, public awareness, saudi arabia, screening practices, visual impairment

## Abstract

Background: Eye diseases, particularly diabetic retinopathy, cataracts, and glaucoma, are significant public health challenges globally, affecting quality of life. Diabetic retinopathy, a common diabetes complication, is a leading cause of visual impairment among working-age adults due to chronic hyperglycemia. Despite treatment advances, awareness of this condition remains low, especially in high-risk populations. This study explores the awareness, knowledge, attitudes, and practices regarding eye health among residents of Jazan City, Saudi Arabia.

Methods: This cross-sectional study utilized an online survey distributed via social media from August 1 to August 30, 2024. Targeting adults aged 18 and older, the survey assessed demographics, knowledge of eye diseases, and awareness of screening practices. After refining through a pilot test and validation by experts, data were collected anonymously via Google Forms (Google, Mountain View, California, United States) and analyzed using IBM SPSS Statistics for Windows, Version 27.0 (Released 2020; IBM Corp., Armonk, New York, United States).

Results: A total of 424 participants completed the survey, comprising 236 females (55.7%) and 186 males (43.9%), with the majority aged 18-45 years (n=379, 89.4%). Significant knowledge gaps were evident, as 284 participants (67%) were unaware of diabetic retinopathy, with only 138 (32.5%) recognizing the condition. Awareness of treatability was limited; 261 participants (61.6%) were uncertain about available treatment options. Social media (n=112, 26.4%) and family or friends (n=106, 25%) were the primary sources of information regarding eye health. While most participants believed in the necessity of regular eye examinations, many had not undergone such screenings.

Conclusion: This study reveals critical gaps in knowledge and awareness of eye diseases, particularly diabetic retinopathy, among residents of Jazan City, Saudi Arabia. Although participants generally recognize the importance of eye health, misconceptions about the causes, risk factors, and treatment options for common eye conditions persist. Targeted public health initiatives and educational interventions are essential to enhance awareness and promote proactive eye health behaviors. Improving understanding and access to eye care services can facilitate the early detection and management of eye diseases, ultimately reducing the burden of visual impairment in the community.

## Introduction

Eye diseases, including diabetic retinopathy, cataracts, and glaucoma, represent significant public health challenges globally, with profound implications for individuals' quality of life and overall health. Diabetic retinopathy, a complication of diabetes mellitus, is a leading cause of visual impairment and blindness among working-age adults [1-3]. It results from microvascular damage due to chronic hyperglycemia, leading to progressive retinal changes that can severely affect vision if not detected and managed promptly. Despite advancements in screening and treatment options, awareness and knowledge of diabetic retinopathy remain suboptimal, particularly in populations at high risk [4,5].

Cataracts, characterized by the opacification of the lens, are another major contributor to vision loss, particularly among older adults. The World Health Organization estimates that cataracts account for approximately 51% of world blindness. Although surgical intervention is highly effective in restoring vision, barriers to timely treatment persist, including a lack of awareness about the condition and its symptoms [2-6].

Glaucoma, often referred to as the "silent thief of sight," encompasses a group of eye disorders that lead to damage of the optic nerve, usually associated with elevated intraocular pressure [3,7]. It is a leading cause of irreversible blindness globally. Many individuals with glaucoma remain undiagnosed until significant vision loss has occurred, underscoring the need for greater awareness and proactive screening.

In Saudi Arabia, the prevalence of these eye conditions is increasing, attributed to rising rates of diabetes, an aging population, and lifestyle changes. However, knowledge and practices regarding regular eye examinations and awareness of eye health issues among the general population are not well documented. This study aims to fill this gap by exploring the awareness, knowledge, attitudes, and practices concerning eye health among residents of Jazan City, Saudi Arabia.

Understanding the awareness and practices surrounding eye health can inform public health initiatives and educational campaigns aimed at promoting the early detection and intervention of eye diseases. Furthermore, identifying potential barriers to eye health education and services can guide healthcare policymakers in developing strategies to improve eye health outcomes within the community.

## Materials and methods

Study design and participants

This cross-sectional study utilized an online survey questionnaire to assess the awareness, knowledge, attitudes, and practices regarding eye health among residents of Jazan City, Saudi Arabia. The target population included adults aged 18 years and older, with no restrictions on gender or educational background. A convenience sampling method was employed to maximize participation, and the survey was distributed via various social media platforms, including WhatsApp, Twitter, and Facebook. This approach aimed to enhance reach and ensure diverse representation within the study population.

Questionnaire development

The survey instrument was developed through a thorough review of existing literature related to eye health awareness and practices. The questionnaire consisted of three main sections. The first section gathered demographic information, including age, sex, education level, occupation, and family history of eye problems. The second section assessed participants' knowledge and awareness of common eye diseases, specifically focusing on diabetic retinopathy, cataracts, and glaucoma, as well as understanding their causes, symptoms, and treatment options. The final section evaluated the participants' awareness of recommended screening practices for diabetic retinopathy and other eye conditions, alongside their sources of knowledge about eye health.

To ensure clarity and relevance, the questionnaire underwent a pilot test involving 30 participants. Feedback was collected to identify ambiguities, leading to necessary revisions based on this input. The final version of the questionnaire was validated for content and face validity by a panel of eye health experts, ensuring that the questions accurately reflected the study's objectives.

Data collection

Data collection occurred from August 1 to August 30, 2024, with the survey remaining accessible online throughout this period. Potential participants were invited to participate through social media postings and direct messages. The survey link was designed to be easily shareable, encouraging participants to forward it to their networks. Prior to participation, informed consent was obtained, ensuring that participants understood the study's purpose, their right to withdraw, and the confidentiality of their responses.

Data analysis

Data were collected anonymously through Google Forms (Google, Mountain View, California, United States) and subsequently exported for analysis using IBM SPSS Statistics for Windows, Version 27.0 (Released 2020; IBM Corp., Armonk, New York, United States). Statistical analyses were performed to evaluate the responses collected. Descriptive statistics, including frequencies and valid percentages, were calculated for demographic variables and survey responses.

## Results

Participant demographics

A total of 424 participants completed the questionnaire. The demographic profile revealed that 236 participants were female (55.7%) and 186 were male (43.9%). The majority of respondents (n=379, 89.4%) were aged between 18 and 45 years, while 39 participants (9.2%) were aged 46-60 years, and four participants (0.9%) were over 60 years of age. Regarding educational attainment, 253 participants (59.7%) held a bachelor's degree, followed by 130 participants (30.7%) with secondary education, and only three participants (0.7%) reported having completed primary education. The occupational distribution indicated that most respondents were students (n=251, 59.2%), with 43 participants (10.1%) unemployed. Furthermore, 279 participants (65.8%) reported a family history of eye problems (Table 1).

**Table 1:** Demographic characteristics of the participants (n=424) Data are presented in frequency (n) and percentages; percentages may not sum to 100 due to rounding.

Variable		Frequency (n)	Percentage (%)
Sex	Female	236	55.7
	Male	186	43.9
Age group (in years)	18-45	379	89.4
	46-60	39	9.2
	Over 60	4	0.9
Educational attainment	Bachelor's degree	253	59.7
	Secondary school	130	30.7
	Vocational/technical	35	8.3
	Primary education	3	0.7
	Intermediate education	1	0.2
Occupation	Student	251	59.2
	Unemployed	43	10.1
	Employee in the educational sector	43	10.1
	Employee in the health sector	42	9.9
	Employee in the military sector	10	2.4
Family history of eye problems	Yes	279	65.8
	No	143	33.7

Knowledge and practices related to diabetic retinopathy

Knowledge regarding diabetic retinopathy among participants demonstrated a substantial gap in awareness. A total of 284 participants (67%) reported that they were not aware of diabetic retinopathy, while 138 participants (32.5%) indicated awareness of the condition. When asked about the understanding of causes related to diabetic retinopathy, 248 participants (58.5%) attributed it to poor blood sugar control, and only 14 participants (3.3%) identified hypertension as a risk factor. Concerning the treatability of diabetic retinopathy, 31 participants (7.3%) believed it was not treatable, while 261 participants (61.6%) were uncertain, and 130 participants (30.7%) affirmed that it was treatable. Screening frequency recommendations showed that 210 participants (49.5%) believed screenings should occur every six months, while 165 participants (38.9%) expressed uncertainty regarding the appropriate timing for screenings (Table 2).

**Table 2:** Knowledge and practices related to diabetic retinopathy (n=424) Data are presented in frequency (n) and percentages; percentages may not sum to 100 due to rounding.

Variable		Frequency (n)	Percentage (%)
Awareness of diabetic retinopathy	Aware	138	32.5
	Not aware	284	67
Understanding of the causes of diabetic retinopathy	Poor blood sugar control	248	58.5
	Uncertain	156	36.8
	Hypertension	14	3.3
	Smoking	4	0.9
Perception of treatability of diabetic retinopathy	Treatable	130	30.7
	Not treatable	31	7.3
	Uncertain	261	61.6
Recommended screening frequency for diabetic retinopathy	Every six months	210	49.5
	Annually	39	9.2
	Only when vision deteriorates	8	1.9
	Uncertain	165	38.9

Sources of knowledge about eye health

The sources from which participants obtained knowledge about eye health varied significantly. A total of 106 participants (25%) reported that family members, friends, or relatives suffering from similar issues provided them with information. Additionally, 112 participants (26.4%) cited social media, books, and television as their sources of knowledge, while awareness campaigns contributed to only 22 participants (5.2%). Healthcare professionals, including doctors and optometrists, provided information for 62 participants (14.6%) (Table 3).

**Table 3:** Sources of knowledge about eye health (n=424) Data are presented in frequency (n) and percentages; percentages may not sum to 100 due to rounding.

Sources of knowledge about eye health	Frequency (n)	Percentage (%)
Family/friend/relative with similar issues	106	25
Healthcare professional (doctor, optometrist)	62	14.6
Social media/books/TV	112	26.4
Awareness campaigns	22	5.2
Other	120	28.3

## Discussion

This study provides valuable insights into the awareness, knowledge, attitudes, and practices related to eye health among residents of Jazan City, Saudi Arabia. The findings highlight significant gaps in awareness, particularly concerning diabetic retinopathy, which is critical given the rising prevalence of diabetes in the region. While a majority of participants reported awareness of common eye diseases, only a minority recognized the risk factors and preventive measures associated with diabetic retinopathy. This discrepancy indicates a pressing need for targeted educational interventions to enhance the understanding of this condition and promote proactive eye health management [5-9].

The study found that approximately 67% of respondents reported a lack of knowledge about diabetic retinopathy. This is concerning, as diabetic retinopathy can lead to irreversible vision loss if not detected early. The low awareness levels are consistent with findings from other studies conducted in similar contexts, suggesting that despite the accessibility of information through healthcare professionals and media, significant barriers remain [4-11]. These may include limited access to eye care services, cultural beliefs, and a general lack of public health campaigns focused on eye health education.

Furthermore, our results revealed that a substantial proportion of participants were unaware of the treatment options available for diabetic retinopathy, with nearly 62% expressing uncertainty about the treatability of the condition. This lack of awareness could potentially deter individuals from seeking timely medical advice and intervention, further exacerbating the public health burden associated with diabetes-related eye diseases [10,12].

The findings also highlight the significant role that family, friends, and social media play in shaping individuals' knowledge about eye diseases. Over a quarter of respondents identified family and friends as their primary source of information, emphasizing the importance of leveraging personal networks in health education strategies. Public health initiatives should consider incorporating peer-led education models and community-based programs to disseminate accurate information regarding eye health, particularly in underserved populations [11-15].

Interestingly, the survey indicated that the majority of participants believed in the necessity of regular eye examinations, with 49% suggesting screenings every six months. However, this belief did not translate into practice, as many reported never having undergone an eye exam. This gap between knowledge and practice suggests that simply increasing awareness may not be sufficient to prompt behavioral change. Barriers such as cost, availability of services, and lack of referral pathways from primary care to eye care specialists need to be addressed to facilitate access to eye examinations [8,12].

This study is subject to several limitations that may impact the generalizability of its findings. Firstly, the use of a convenience sampling method may introduce selection bias, as participants who are more engaged with social media or have a higher interest in health issues may have been overrepresented. Additionally, the reliance on self-reported data may lead to inaccuracies in participants' responses regarding their knowledge and practices related to eye health. Furthermore, the cross-sectional design limits the ability to infer causal relationships between awareness and health behaviors. Finally, the study was conducted in a specific geographic area, which may limit the applicability of the results to other regions of Saudi Arabia or different cultural contexts. Future research should employ a more diverse sampling strategy and longitudinal designs to validate and expand upon these findings.

## Conclusions

This study highlights critical gaps in knowledge and awareness of eye diseases, particularly diabetic retinopathy, among residents of Jazan City, Saudi Arabia. Despite a general acknowledgment of the importance of eye health, significant misconceptions persist regarding the causes, risk factors, and treatment options associated with common eye conditions. To address these gaps, targeted public health initiatives and educational interventions are essential to promote awareness and encourage proactive eye health behaviors. By improving knowledge and access to eye care services, we can enhance the early detection and management of eye diseases, ultimately reducing the burden of visual impairment in the community.
